# Applying causal mediation methods to clinical trial data: What can we learn about why our interventions (don't) work?

**DOI:** 10.1002/ejp.964

**Published:** 2016-10-14

**Authors:** R. Whittle, G. Mansell, P. Jellema, D. van der Windt

**Affiliations:** ^1^ Research Institute for Primary Care & Health SciencesKeele UniversityStaffordshireUK; ^2^ Department of Paediatric OncologyEmma Children's HospitalAcademic Medical CenterAmsterdamThe Netherlands

## Abstract

**Background:**

Many randomized controlled trials (RCTs) of psychosocial interventions for low back pain (LBP) have been found to have only small effects on disability outcomes. Investigations of the specific mechanisms that may lead to an improvement in outcome have therefore been called for.

**Methods:**

We present an application of the causal inference approach to mediation analysis using the example of a cluster RCT in a primary care population with (sub)acute LBP randomized to either usual GP care (*n* = 171) or a minimal psychosocial intervention (*n* = 143). Mediation analysis explored the causal pathway between treatment allocation and disability at 3 months by considering pain catastrophizing, fear‐avoidance beliefs, distress and receiving and following advice as potential mediators, all measured at 6 weeks. We have attempted to explain this approach to mediation analysis in a step‐by‐step manner to help clinical researchers apply this method more easily.

**Results:**

In unadjusted mediation analyses, fear‐avoidance beliefs were identified as a mediator of treatment on disability, with an indirect effect of −0.30 (95% CI: −0.86, −0.03), although this relationship was found to be non‐significant after adjusting for age, gender and baseline scores. This finding supports the trial authors’ hypothesis that while fear‐avoidance beliefs are important, this intervention may not have targeted them strongly enough to lead to change.

**Conclusion:**

The use of mediation analysis to identify what factors may be part of the causal pathway between intervention and outcome, regardless of whether the intervention was successful, can provide useful information and insight into how to improve future interventions.

**Significance:**

This study presents a step‐by‐step approach to mediation analysis using the causal inference framework to investigate why a psychosocial intervention for LBP was unsuccessful. Fear‐avoidance beliefs were found to mediate the relationship between treatment and disability, although not when controlling for baseline scores.

## 1 Introduction

Many randomized controlled trials (RCTs) have been conducted aiming to reduce disability in patients with low back pain (LBP) using psychosocial interventions (see Kent and Kjaer, 2012; Pincus and McCracken, 2013). Identifying what specific treatment components or mechanisms may lead to an improvement in outcome are required (Thorn and Burns, 2011; Ehde et al., 2014).

A mediating factor is one that helps to explain how a treatment works (Vlaeyen and Morley, 2005). Mediation analysis is increasingly used in RCTs to confirm the hypothesized working mechanism underlying an intervention, or examine why an intervention was not successful by helping to identify possible improvements for the design and evaluation of future intervention studies.

Jellema et al. (2005b) conducted a cluster RCT which compared a minimal psychosocial intervention strategy (MIS) with usual General Practitioner (GP) care in a primary care population with (sub)acute LBP (Jellema et al., 2005b). The MIS aimed to identify psychosocial prognostic factors (specifically fear‐avoidance beliefs, catastrophizing thoughts and distress) so that they could be discussed during the consultation. GPs were trained to firstly explore the presence of psychosocial factors using standardized questions; they then gave the patient information about available treatments for back pain, focusing on addressing the psychosocial factors identified; specific goals to resume normal activities were then set between the GP and patient, and the patient was given a copy of the Back Book which served to reinforce the information given by the GP. The authors hypothesized that patients in the intervention arm would report better functioning and perceived recovery and less time off work due to their LBP than patients in the usual care arm, but found that the intervention was no more effective than usual care. Post‐hoc analyses suggested that although GPs provided the right behavioural messages and were able to identify psychosocial risks, the intervention was not sufficient to modify patients’ behaviours or shift risk factors (Jellema et al., 2005a).

One key issue across all mediation literature is the importance of a theoretical basis underlying the proposed mediation pathway (Mathieu et al., 2008; Green et al., 2010; MacKinnon and Pirlott, 2015). It has been argued that a sound theoretical basis can help select which potential mediators to focus the analysis on (Murphy et al., 2009), and also strengthen the argument for causality by showing how variables may occur in a particular order (Mathieu and Taylor, 2006). Use of a theoretical model allows predefined mediation pathways to be tested by allowing the a priori identification of key mediating factors. Several of the factors included in this study (catastrophizing, fear‐avoidance beliefs and disability) are key aspects of the fear‐avoidance model (Vlaeyen and Crombez, 1999; Vlaeyen and Linton, 2012) which hypothesizes that the experience of pain leads to catastrophizing thoughts in some patients, resulting in avoiding activities they feel may lead to further damage, leading to disability (Vlaeyen and Crombez, 1999).

The present study extends the findings of Jellema et al. by conducting formal mediation analyses via the causal inference approach to investigate the assumed causal pathways underlying the intervention. We tested whether key factors of interest to the trial authors had an indirect (mediating) effect on the effect of treatment on disability outcome.

## 2 Methods

### 2.1 Background to mediation analysis

**Figure 1:**
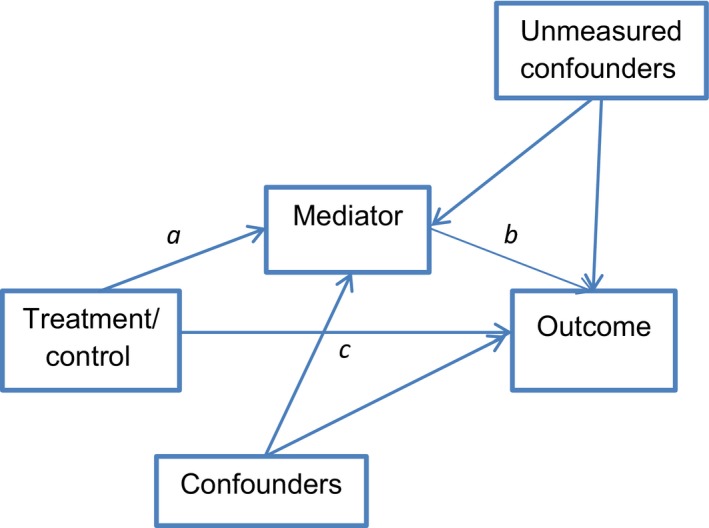
Example mediation model.

Mediation analysis has become increasingly popular over the last 30 years, with many studies in the psychological literature basing their analyses on the causal steps approach by Baron & Kenny (Baron and Kenny, 1986) or the product of coefficients approach (MacKinnon et al., 2007). However, these approaches, based on linear regression, make strong assumptions about the absence of any confounding variables between each part of the model (Imai et al., 2011; Valeri and Vanderweele, 2013), with Baron and Kenny ignoring the issue of confounding. This is a problem even in RCTs, as while path *a* (see Fig. 1) and the total effect (*ab* + *c*) can be assumed to be free of confounding as a result of randomization, path *b* may contain hidden confounders or additional explanatory variables because both the mediator and outcome are outcomes of randomization (Imai et al., 2011; Valeri and Vanderweele, 2013), which may be important in explaining the mediating pathway. These assumptions are difficult to relax, as it is very difficult to account for all potential confounding and additional potential mediating variables in a single model.

Further development in mediation analysis techniques has taken place in the statistical and epidemiological literature following the introduction of the causal inference approach via the potential outcomes framework. This framework describes how we only ever see one outcome for each person, i.e. for those in the treatment group of an RCT, we know their outcome following treatment but will never know what their outcome would have been had they been randomized to the control group (Imai et al., 2011; Dunn et al., 2013). The outcome that cannot be observed is denoted as the counterfactual. So we can think of the treatment effect as the difference between the two potential values of the outcome. As both outcomes cannot be observed in any one individual, we cannot observe the individual treatment effects, but because of random allocation we can estimate the average treatment effect (i.e. the efficacy of the intervention compared to the control) (Dunn et al., 2013). In mediation analysis, we are assuming that there is an effect of treatment on a mediating variable (*a* path, Fig. 1), which in turn leads to an effect on the outcome (*b* path, Fig. 1), and we can then similarly estimate the average treatment effects on the outcome through the mediator (*ab* path, Fig. 1; Dunn et al., 2013). There are several differences between this counterfactual approach to mediation analysis and traditional regression approaches. Firstly, statistical approaches to mediation analysis using regression assume linear effects and no interactions, whereas the counterfactual approach allows for the estimation of direct and indirect effects when there are interactions (Valeri, 2012). Secondly, linear regression approaches do not discuss identifiability assumptions (conditions needed for the results of statistical procedures to have a causal interpretation). Thirdly, the counterfactual approach allows for the use and interpretation of binary and categorical outcomes and/or mediators. The counterfactual approach is also more able to explicitly acknowledge the effect of potential confounding factors.

It is important to note that while different approaches to mediation analysis have been developed with varying language and terminology, the underlying aim of identifying indirect effects is the same.

### 2.2 Description of the mediation model

The basic mediation model is shown in Fig. 1. The indirect (mediating) effect is represented by the path from treatment to outcome via the mediator (paths *a* and *b*), and the direct effects are represented by each of the *a, b* and *c* paths separately.

**Direct effects** are the influence on the outcome, or on the mediator, that is not mediated by other variables in the model (Pearl, 2001) (i.e. the change in the outcome (path *c* and path *b*) or the change in the mediator (path *a*) that occurs from the change in the intervention (path *a* and path *c*) or from the change in the mediator (path *b*) when all the other variables in the model are fixed).

An **indirect (mediated) effect** (*ab* path, Fig. 1) expresses the portion of the intervention effect that is mediated through a specific mediator. This is estimated by how much the outcome would change if everyone in the study had the intervention and the mediator changed from its natural level had each individual been assigned to control, to its natural level had each individual been assigned to treatment.

An indirect effect can still be present if there is no direct effect of treatment on outcome (i.e. no treatment effect) (Collins et al., 1998). *‘Mediating effect’*, a term from the psychological literature, and ‘*indirect effect’*, a term from the epidemiological literature, are often used synonymously. However, there have recently been calls to distinguish between the two, with ‘mediating effect’ being reserved for situations in which research designs that better allow for causal inference are employed (Kline, 2015). For the purpose of this paper, we will assume that mediating and indirect effects are the same.

The **total effect** is how much the outcome would change overall if treatment group was changed from the control to the intervention, which is the sum of the direct and indirect effects (*ab* + *c*).

### 2.3 Example: low back pain trial

#### 2.3.1 Design and study population

The study population comprised of 314 participants from a cluster RCT (Jellema et al., 2005b) which randomized patients to either usual care (*n *=* *171) or a minimal intervention strategy (*n *=* *143). Patients were recruited by one of 60 general practitioners in 41 general practices in the Netherlands (20 practices with 28 GPs were randomized to the intervention, and 21 practices with 32 GPs were randomized to usual care). To be included in the study, patients needed to be aged between 18 and 65 years with non‐specific low back pain of less than 12 weeks duration or a worsening of existing low back pain, and were excluded if their pain was the result of specific spinal pathology or if they were pregnant.

#### 2.3.2 Measures

All measures were recorded by patient completed questionnaires.

Disability score at 3 months, measured by the Roland‐Morris Disability Questionnaire (RMDQ) (Rolland and Morris, 1983) was defined as the outcome for this study. The RMDQ is measured on a 24‐point scale with greater values indicating greater disability.

Factors that were considered as potential mediating variables between treatment and disability were pain catastrophizing, fear‐avoidance beliefs, distress and the advice provided by GPs to rest when pain increases (hypothesized to be lower in the minimal intervention strategy group), to gradually increase exercise regardless of pain, or to stay active and moving regardless of pain (hypothesized to be more frequent in the minimal intervention strategy group). Fear‐avoidance beliefs, pain catastrophizing and distress were investigated because these were factors that the GPs explicitly set out to identify and target during the intervention. Advice was investigated as we felt that whether patients reported that they received and followed particular advice given by the GP in the information phase of the MIS may also have led to changes in outcome.

Pain catastrophizing was measured using the catastrophizing subscale of the coping strategies questionnaire (CSQ; Harland and Georgieff, 2003), composed of six items that assess negative thoughts related to pain as well as catastrophic thoughts and ideations about pain (range 0–36). Higher scores reflect worse catastrophizing.

Fear‐avoidance beliefs were measured using the fear‐avoidance beliefs questionnaire (FABQ; Waddell et al., 1993; range 0–24). A higher score indicates stronger fear‐avoidance beliefs.

Distress was measured using the distress subscale from the Four Dimensional Symptom Questionnaire (4DSQ; Terluin et al., 2006), containing 16 items (range 0–32). A higher score indicates more severe distress.

Pain catastrophizing, fear‐avoidance beliefs and distress were measured at baseline and 6 weeks. Receiving and following advice to rest when pain increases, to gradually increase exercise regardless of pain, or to stay active and moving regardless of pain were given as Yes/No responses at the 6 week follow‐up point.

### 2.4 Statistical analysis

The analysis was carried out in several stages, firstly using descriptive statistics to describe the sample then testing the direct effects of the intervention on each of the potential mediators (the *a* path of Fig. 1). We then fit mediation models to estimate the direct and indirect effects. Specifically, we tested five models (to test each individual potential mediator), firstly assuming no confounding and then adjusting for potential confounding factors. It is likely that other, unmeasured confounders could be present along the *b* pathway, but in this analysis we must assume that the model only includes the specified paths. All analyses were conducted using Stata/MP 13.1 (Stata Corporation, College Station, TX, USA).

#### 2.4.1 Step 1. Descriptive statistics

Median (IQR) values were calculated for the continuous variables (catastrophizing, distress, fear‐avoidance beliefs and disability score) at baseline and at 6 weeks for the mediators and at 3 months for the outcome (disability score). The frequencies of the binary measures (received and followed advice to rest, received and followed advice to keep active, or received and followed advice to gradually increase exercise) were tabulated by treatment.

#### 2.4.2 Step 2. Test of direct effect of treatment on the mediator (*a* path, Fig. 1)

Univariable linear regression models were fitted to the potential mediators to test whether there was an association between treatment and the mediator. Since a variable can only be a mediator of treatment if there is a significant effect (*p* < 0.05) of treatment on the mediator (path *a*), mediation models in Step 3 were only fitted to variables that were significantly associated with treatment.

#### 2.4.3 Step 3. Test of the indirect (mediating) effect (*ab* path, Fig. 1)

Mediation analysis was performed using the methods described by Valeri and Vanderweele, 2013 to investigate direct and indirect effects of the minimal intervention strategy on disability at 3 months.

The –paramed‐ command (based on the SAS and SPSS macros by Valeri and Vanderweele, 2013) performs the analysis by fitting a regression model to the outcome (linear regression in this study due to it having a continuous outcome, but the command can also be used to fit a logistic, log linear, Poisson or Negative binomial regression model dependent on the outcome), with treatment and the current mediator included as covariates, and then fitting a regression model to the mediator (linear or logistic depending on the mediator) including treatment as a covariate. The direct and indirect effects are then calculated from the coefficients of these models, and the confidence intervals of the effects are calculated either by the delta method (Sobel test) or using bootstrap replications (1000 by default). In this example, 1000 bootstrap replications were performed to provide a more accurate estimate of the confidence interval of a non‐normally distributed indirect effect and to account for non‐normality of the mediators and outcome variable. As described above, we are looking at the portion of the intervention effect (or absence of) that is mediated through a specific mediator. Each mediation model used the 6 week score for each potential mediator and the 3 month outcome of disability.

#### 2.4.4 Step 4. Testing of the indirect (mediating) pathway for potential confounding

As treatment was randomly allocated, the baseline characteristics of the two treatment groups were similar, but estimates of the direct and indirect effects can be biased even in randomized trials when there are unmeasured confounders between the mediator and outcome (*b* path, Fig. 1; Emsley et al., 2010). Hence, we performed the mediation analysis with and without adjustment for age, gender and baseline measures of the outcome and mediators (only possible for catastrophizing, fear avoidance beliefs and distress), attempting to control for these as potential confounders in order to add robustness to our analysis.

## 3 Results

### 3.1 Step 1. Descriptive statistics

As was seen in the original trial (Jellema et al., 2005b), there was very little difference in the change in disability, catastrophizing, distress and fear‐avoidance beliefs between the two treatment groups (Table 1). There was a large difference in the percentage of patients who received and followed certain advice between the treatment groups, as would be expected as this was something that was targeted in the minimal intervention strategy. For example, in the minimal intervention strategy group, 80% of people received and followed advice to stay active and moving regardless of pain, whereas in the usual care group only 8% of patients received and followed this advice.

**Table 1:** Baseline characteristics, 6‐week scores for potential mediator and 3‐month outcome by treatment group

	Usual care (*n* = 171)	Minimal intervention strategy (*n* = 143)
Male; no. (%)	90 (52.6)	75 (52.5)
Age; mean (SD)	42.0 (12.0)	43.4 (11.1)
Disability (0–24)		
Baseline; Median (IQR)	13 (8, 16)	13 (7, 16)
Three months; Median (IQR)	2 (0, 5)	2 (0, 6)
Catastrophizing (0–36)		
Baseline; Median (IQR)	11 (6, 15)	10 (5, 15)
Six weeks; Median (IQR)	7 (3, 12)	7 (3, 13.5)
Distress (0–32)		
Baseline; Median (IQR)	8 (4, 14)	6 (3, 12)
Six weeks; Median (IQR)	3 (0, 9)	3 (0, 8)
Fear‐avoidance beliefs (0–24)		
Baseline; Median (IQR)	15 (12, 19)	15 (11, 18)
Six weeks; Median (IQR)	13 (10, 17)	12 (7, 16)
Received and followed advice to		
Rest when pain increases; no. (%)	81 (47.4%)	42 (29.4%)
Stay active and moving regardless of pain; no. (%)	14 (8.2%)	115 (80.4%)
Gradually increase exercise regardless of pain; no. (%)	32 (18.7%)	93 (65.0%)

### 3.2 Step 2. Test of direct effect of treatment on the mediator

This step tested the *a* path in the mediation model (Fig. 1), to identify which potential mediators had a statistically significant association with treatment allocation. The potential mediators which were significant (*p* < 0.05) within the regression models (Table 2), were fear‐avoidance beliefs, received and followed advice to keep active and moving, and received and followed advice to gradually increase exercise. On average, fear‐avoidance beliefs score at 6 weeks was 1.36 (95% CI 0.10, 2.62) units lower in the intervention group than in the control group, suggesting that the intervention had a positive impact on fear‐avoidance beliefs.

**Table 2:** Regression models testing association between treatment and potential mediators

Potential mediator (measured at 6 weeks)	Estimate (95% CI)	*p*‐Value
Catastrophizing (range: 0–36)	0.42 (−1.12, 1.97)^a^	0.589
Distress (range: 0–32)	−0.09 (−1.61, 1.44)^a^	0.913
Fear‐avoidance beliefs (range: 0–24)	−1.36 (−2.62, −0.10)^a^	0.034
Advice to rest	1.06 (0.64, 1.76)^b^	0.826
Advice to exercise	5.57 (3.26, 9.50)^b^	<0.001
Advice to stay active	2.90 (1.48, 5.68)^b^	0.002

a Coefficient (95% confidence interval) from linear regression model.

b Odds Ratio (95% confidence interval) from logistic regression model.

### 3.3 Step 3. Test of the indirect (mediating) effect

From the analyses presented above, the only potential mediator with a significant indirect effect (unadjusted) between treatment and disability was fear‐avoidance beliefs. The indirect, direct and total effects of each of the models are given in Table 3. The indirect effect (*ab* path, Fig. 1) of treatment on disability at 3 months, with fear‐avoidance beliefs as the potential mediator, was −0.30 (95% CI: −0.86, −0.03). This means that if everyone in the study had the intervention, the 3‐month disability score would reduce on average by 0.30 units (indirect effect from Table 3) if each participant's fear‐avoidance beliefs reduced by the mean difference in fear‐avoidance beliefs between the control and intervention group (1.36, as given in Table 2). No significant interaction term was found within the models in our study.

**Table 3:** Indirect, direct and total effects of the mediation models on disability outcome at 3 months

Mediator		Indirect (mediating) effect	Direct effect of treatment on outcome	Total effect
Fear‐avoidance beliefs (*N* = 290)	Unadjusted	−0.30 (−0.86, −0.03)	0.74 (−1.26, 2.94)	0.44 (−1.46, 2.37)
	Adjusted for age and gender	−0.29 (−0.89, −0.03)	0.74 (−1.20, 2.90)	0.45 (−1.43, 2.45)
	Adjusted for age, gender and baseline mediator and outcome scores	−0.18 (−0.65, 0.00)	0.62 (−1.47, 2.91)	0.44 (−1.80, 2.55)
Advice to exercise (*N* = 262)	Unadjusted	0.06 (−0.72, 0.63)	−0.27 (−1.82, 1.10)	−0.21 (−2.14, 1.20)
	Adjusted for age and gender	0.08 (−0.66, 0.65)	−0.32 (−1.88, 1.06)	−0.24 (−2.17, 1.22)
	Adjusted for age, gender and baseline outcome score	−0.12 (−1.00, 0.46)	−0.08 (−1.56, 1.28)	−0.20 (−2.20, 1.14)
Advice to stay active (*N* = 267)	Unadjusted	0.11 (−0.14, 0.55)	−0.49 (−2.76, 1.00)	−0.37 (−2.20, 1.08)
	Adjusted for age and gender	0.09 (−0.16, 0.54)	−0.48 (−2.65, 1.01)	−0.39 (−2.21, 1.04)
	Adjusted for age, gender and baseline outcome score	0.04 (−0.21, 0.42)	−0.37 (−2.52, 1.04)	−0.32 (−2.20, 1.04)

### 3.4 Step 4. Test of the indirect (mediating) pathway for potential confounding

When adjusting for baseline values of disability and fear‐avoidance beliefs, the indirect effect of fear‐avoidance beliefs on 3‐month disability was no longer statistically significant (indirect effect −0.18, 95% CI −0.65 to 0.00), suggesting that baseline values of both fear‐avoidance beliefs and disability may potentially play a role in the hypothesized mediating pathway (Table 3).

## 4 Discussion

This study involved further analysis of a well‐conducted RCT comparing the effectiveness of a GP‐delivered minimal intervention strategy with usual care. The original RCT found the intervention to be no more effective than usual care, and subsequent analyses by the trial authors found that this may be due to the intervention only being effective within a subset of participants or because the variables being targeted simply were not being targeted strongly enough. The present analysis investigated whether key psychosocial factors and activity behaviours hypothesized to be addressed by the intervention had an indirect effect on the effect of treatment on outcome. None of the potential mediators were found to significantly mediate outcome in this population. Fear‐avoidance beliefs had an indirect effect on the relationship between treatment and disability, but after adjusting for age, gender and baseline scores of fear‐avoidance beliefs and disability, this effect was no longer present, hence we cannot say that fear avoidance beliefs mediated the relationship between treatment and disability.

It is interesting that while advice to keep active and to gradually increase physical activity were potential mediators of outcome in that they were associated with treatment, they were not associated with improvement in the outcome of disability and hence were not mediators of the relationship between treatment and outcome. One explanation could be that while participants received the advice and reported behaviour change, they may not have sufficiently changed their behaviour and the self‐reported scores perhaps reflect a social desirability bias. This intervention was designed to be ‘minimal’ and it might be that a more intensive intervention is required to actually change patient behaviour. The finding that fear‐avoidance beliefs did have an indirect effect on the relationship between treatment and outcome (although not once the model was adjusted) suggests that this variable is potentially a target for intervention, but that the intervention either did not reduce fear‐avoidance beliefs enough for it to be an effective treatment, or there were other factors explaining the large reduction in disability. For example, as both the intervention and control arms improved fairly equally, the original results could reflect the natural history of an episode of back pain, particularly in a patient group that included people presenting to their GP with an exacerbation of pain (an inclusion criteria of the original study) and people who had been experiencing pain for a relatively short period of time (around 12 days; see Jellema et al., 2005b).

This analysis did not find any statistically significant mediating effects in the variables tested, although fear avoidance beliefs were identified as having a significant indirect effect before adjustment for baseline scores. The results of analyses that do not adjust for confounders should not be over interpreted. However, the absence of statistically significant mediating effects identified could be due to the study being underpowered to detect these effects, as the original RCT was not powered for this analysis. When designing RCTs, the sample size needed to investigate mediating mechanisms should be considered.

The use of mediation analysis to identify what factors may be part of the causal pathway between intervention and outcome, regardless of whether the intervention was successful or unsuccessful, can provide useful information and insight into how to improve future interventions. Mediation analysis provides a more robust test of what might be responsible for an association (or lack of an association) between intervention and outcome than simply looking at separate associations between the three variables as in other common approaches to mediation analysis. Importantly, when a theoretical model is used to underpin the intervention, predefined hypotheses for how the intervention is thought to work are in place to be tested in a mediation analysis.

We used the causal inference framework to perform this mediation analysis, but as highlighted in the introduction this is not the only way to investigate mediating variables. The method chosen depends on the study design, the data itself and the specific mediation question to be answered. Preacher (2015) describes the approaches from psychology as focusing more on the design of the study, and the causal inference methods from statistics as focusing more on the formal criteria necessary to infer causality. The use of longitudinal data collection and analysis methods that can readily handle longitudinal data, such as structural equation modelling (SEM; e.g. Cook et al., 2006) and latent growth curve modelling (LGM; e.g. Cheong et al., 2003; Roesch et al., 2009) are frequently found in the psychological literature to help account for temporality, a key aspect of establishing causality. In the case of a continuous mediator and continuous outcome, the indirect effects in both the traditional regression and the causal inference approaches are equivalent, but the causal inference approach can be extended, without the necessity of standardization, to include binary mediators and/or outcomes. SEM and LGM methods extend the use of linear regression analysis to include latent factors; latent constructs in the case of SEM, and the incorporation of time in the case of LGM. Both methods allow the use of change scores or numerous time points, a design issue thought to help solve the issue of temporality by being able to more fully explore when the mediator and outcome change (Kazdin, 2007). This is not something that can currently be incorporated into causal inference methods. However, it is acknowledged that there are other important aspects of causality that need to be addressed in order to truly establish whether a causal link can be confirmed, such as the absence of other potential alternative explanations for the observed link (Shadish et al., 2002). It is these aspects that the causal inference literature attempts to target (Valeri and Vanderweele, 2013). Preacher (2015) concludes that no method is superior and that each has strengths and limitations, for example, the ‐paramed‐ command used in our analyses cannot adjust for clustering, which was present in this dataset. For the purpose of the illustrative example given here, we have not accounted for any clustering which may be present to enable us to clarify our main points concerning mediation analysis. We have also not accounted for any potential correlation between the mediators. The method chosen to perform mediation analysis should depend on the study design and available measures of the mediator and outcome variable(s). Certainly, methods from both the psychological and statistical disciplines appear to be converging (Preacher, 2015); macros for statistical software originally developed in the psychological literature (Preacher and Hayes, 2004) have been extended to incorporate non‐linear variables; Imai et al. (2010) agree that the product of coefficients approach, a frequently used psychological approach to mediation, is appropriate provided certain assumptions are met; and others have presented suggestions of how to integrate SEM models and longitudinal designs with the causal inference literature (De Stavola et al., 2015; Muthén and Asparouhov, 2015; Bind et al., 2016). This suggests that the methods used to carry out mediation analysis are becoming more ‘generic’ and applicable to a wider number of designs.

We have presented an example of how mediation analysis can be used to investigate why an intervention does or does not work. The example uses a specific methodology and framework (causal inference via potential outcomes) not often applied to clinical data, to highlight how the analysis of mediators is moving forward and gaining complexity in the effort to identify causal links. We have attempted to explain this method in a simple, step‐by‐step approach to help increase its uptake within the applied literature. Mediation analysis is a valuable tool available to researchers and clinicians in that it allows us to move beyond the “efficacy” question in clinical trials and investigate how an intervention worked. This then provides important information for future interventions in terms of what components might be most useful to include and what can perhaps be removed, therefore improving the efficiency of treatments.

## Author contributions

P.J. conceived and carried out the original trial; D.v.d.W. conceived the idea for the present analysis; R.W. completed the statistical analysis; G.M., D.v.d.W. and R.W. drafted the thesis. All authors discussed the results, commented on the manuscript and approved the final version of the manuscript.
